# Epilepsy and Deep Brain Stimulation of Anterior Thalamic Nucleus

**DOI:** 10.7759/cureus.18199

**Published:** 2021-09-22

**Authors:** Carlos D Perez-Malagon, Miguel A Lopez-Gonzalez

**Affiliations:** 1 Anatomy, Centro de Ciencias Biomedicas, Universidad Autonoma de Aguascalientes, Aguascalientes, MEX; 2 Neurosurgery, Loma Linda University Medical Center, Loma Linda, USA

**Keywords:** surgery, neuromodulation, anterior thalamic nucleus, deep brain stimulation, epilepsy

## Abstract

Presently, at least 60 million people are suffering from epilepsy worldwide. Although multiple pharmacological options for treatment exist, about 30% to 40% of these patients are estimated to have drug-resistant epilepsy (DRE), which is associated with severe disability and morbidity. The surgical treatment options are restricted to either open surgical procedures or laser ablations. When a resective option is not favorable, then neuromodulation options such as vagal nerve stimulation and deep brain stimulation are considered. A relatively recent and more commonly used clinical application is the deep brain stimulation (DBS) of the anterior thalamic nucleus, FDA approval for which was obtained in 2018. Furthermore, new technological advances in DBS technology are expected to positively impact the treatment options of these patients.

## Introduction and background

Not all patients with medically refractory epilepsy are suitable candidates for surgical resection intervention. Additionally, although some patients may be suitable candidates for resective surgery, they are sometimes reluctant to undergo surgery owing to the invasiveness and the associated risk factors. Other patients do not achieve long-term relief from seizures with surgery. In such cases, neuromodulation of the nervous system, such as deep brain stimulation (DBS), may be an ideal treatment option. Thus, the current review discusses epilepsy and its surgical management from the neuromodulation perspective.

## Review

The word epilepsy is derived from the Greek word epilambanein, which means “to be seized.” Epilepsy has existed from time immemorial, with written evidence of it available in the form of a 4000-year-old Akkadian tablet. Discovered in Mesopotamia, the tablet has the following inscription, which describes a seizure: “His neck turning left, hands and feet are tense, and his eyes wide open, and from his mouth, froth is flowing without him having any consciousness” [1]. Many years later, Hippocrates attributed epilepsy to the brain and suggested that it was hereditary rather than contagious. He knew that epilepsy had its origin in the brain and is related to traumatic seizures, and the early surgeons treated those subjects by trephination [2]. Aretaeus of Cappadocian in the second century also recommended trephination as a treatment for epilepsy.

The modern understanding and treatment of epilepsy started in the 20th century, and it was the late 20th century when several international societies were formed to gain more knowledge on epilepsy.

An epileptic seizure was defined conceptually by the International League Against Epilepsy (ILAE) as a transient occurrence of signs and/or symptoms due to abnormal excessive or synchronous neuronal activity of the brain. Additionally, epilepsy is defined as a disorder of the brain characterized by an enduring predisposition to generate epileptic seizures, and by the neurobiologic, cognitive, psychological, and social consequences of this condition. It is important to consider that not all seizures are considered epilepsy when nearly 10% of people worldwide can have one seizure during their lifetime. The definition of epilepsy requires the occurrence of at least one epileptic seizure [3,4]. On the other hand, also per ILAE, the practical clinical definition of epilepsy should have any of the following conditions: 1) At least two unprovoked seizures occurring > 24 hours apart; 2) One unprovoked seizure and a probability of further seizures similar to the general recurrence risk (at least 60%) after two unprovoked seizures, occurring over the next 10 years; 3) Diagnosis of an epilepsy síndrome [5].

Epidemiology

Epilepsy is a common chronic neurological disorder with recurrent episodic attacks, epileptic seizures, and their somatic and psychiatric consequences that affects around 60-70 million people of all ages, races, social classes, and geographical locations. It affects the social, behavioral, and economic conditions of the patient and their families. Nearly 80% of these affected individuals live in low- and middle-income countries [6,7].

It s estimated that 1.2% of the U.S. population has epilepsy, and about one-third of patients have refractory epilepsy (i.e., seizures not controlled by two or more appropriately chosen antiepileptic medications or other therapies), which is also known as drug-resistant epilepsy (DRE) [3].

The lifetime prevalence in the general population varies between 2.3 to 15.9 per 1,000 in high-income countries and between 3.6 to 15.4 per 1,000 in low-income countries [7]. In a meta-analysis including incidence studies of epilepsy, the pooled incidence rate was 61.4 per 100,000 person-years (95% CI 50.7-74.4). However, epilepsy has a bimodal distribution according to age with peaks in the youngest individuals and the elderly. In adolescence, it is 21/100,000, during adulthood 35/100,000, and in elderly people, it is > 60/100,000 [7-9].

Mortality in patients with epilepsy increases three times up to 1/1000, being higher in low- and middle-income countries and rural areas [6,10].

The sudden death of patients with epilepsy with rare seizures is due to status epilepticus, physical injuries, and drowning. However, for those that have not controlled frequent seizures and have DRE, the risk increases 15-fold (18/1000) [6,7,11]. The incidence of death is 1.2 per 1,000 person-years (95% CI 0.9-1.5). It is higher in men (1.41) than in women (0.96). Sudden death accounted for 5.2% of all deaths and 36% of deaths in the 0-15 years age group [12].

Pathophysiology

A seizure seems to be produced when the normal balance between excitation and inhibition in the brain is disturbed. This imbalance results from alterations at many levels of brain function such as genetic and subcellular signaling cascades to neuronal circuits [3].

In the mechanism of epilepsy, several neurotransmitters such as serotonin, dopamine, gamma-aminobutyric acid (GABA), glutamate, and noradrenaline are involved. However, GABA and glutamate are the most studied. It is known that neuronal hyperexcitability is caused by variations in GABA-mediated inhibition as well as in glutamate-mediated excitation [13].

The causes of epilepsy can be classified as structural, genetic, infectious, metabolic, immune, and unknown causes. The most common specific abnormalities are brain damage from prenatal or perinatal causes, brain malformations, acquired factors such as trauma, strokes, and infections (meningitis, encephalitis, or neurocysticercosis), certain genetic syndromes, prolonged symptomatic seizures, neurodegenerative diseases, and tumors. Several series have reported that only 40 out of 100 cases of epilepsy have a clear known etiology [13].

An expert consensus of the International League Against Epilepsy (ILAE) has divided the disease into four syndromes: 1) Generalized epilepsy syndromes, with presumed polygenic etiologies, 2) Focal epilepsy syndromes with genetic, structural, or genetic-structural etiologies, 3) Combined generalized and focal epilepsy syndrome with polygenic etiology, 4) Specific group of developmental and/or epileptic encephalopathies.

The pathophysiology rationale for deep brain stimulation (DBS) in epilepsy is based on the hypothesis for movement disorders with potential cellular inhibition or excitation at the target location [14]. It is proposed that stimulation of directs targets or networks will either help to disrupt seizure propagation or raise the seizure threshold by suppressing neuronal circuits that favor seizure emergence. Low-frequency stimulation has been shown to restore neuronal electrical activity, while high-frequency stimulation can be more effective in disrupting the propagation of synchronous neuronal activity [15].

Animal studies have supported the role of the Papez circuit in seizure occurrence, which links hippocampal projections through the fornix and mammillary nucleus to the anterior thalamic nucleus (ANT). These projections radiate from ANT to the cingulum, then parahippocampus and returning to the hippocampus. Disruptions in the Papez circuit have been observed in different types of epilepsy [16]. Additionally, given the presence of projections from the cerebellum, subthalamic nucleus, and centromedian nucleus of the thalamus to the Papez circuit, these all have been considered as potential DBS targets for epilepsy [17].

Clinical features

Seizures can be either convulsive or non-convulsive. The convulsive type accounts for about 60% of the cases, whereas the remaining 40% of the seizures are non-convulsive, such as absence seizures. However, the frequency of these seizures varies in different series. Seizures can be as frequent as less than one per year to several per day [18].

Convulsive seizures are produced by the sudden occurrence of electrical activity in the brain due to abnormal discharges or the hyperexcitability of neurons with synchronicity and an imbalance between excitation and inhibition. It originates from different sites of the brain, leading to abnormal involuntary muscle contractions that may involve the body partially or entirely and can lead to damage to the brain or other parts of the body. Sometimes, seizures are accompanied by loss of consciousness and the inability to control bowel or bladder function. Occasionally, a single seizure can cause changes in neural development, thereby producing behavioral and cognitive changes [3,13].

Seizures can be accompanied by momentary symptoms such as loss of awareness or consciousness, and disturbances in movement, vision, hearing, and taste, diverse sensations, and changes in mood and cognitive functions.

Patients develop a “postictal state,” which occurs from the end of an epileptic seizure and last until the individual returns to a normal neurological state. The type, intensity, and duration of the postictal state’s symptoms, such as depression, anxiety, irritability, hypersalivation, euphoria, hypomania, sleep, coma, lethargy, fatigue, vomiting, anorexia, laughter, and sighing, vary significantly [19].

Diagnosis

Significant variations in its correct diagnosis and adequate treatment can be observed worldwide ranging from 10% in developed countries to 75% in low-income countries, which leads to approximately 0.3% of deaths [13]. There is no single specific test to diagnose epilepsy. A complete history and a complete neurologic examination are the cornerstones of the diagnosis of seizures and epilepsy, while laboratory tests serve as adjunctive tests. A diagnosis of epilepsy is generally based on electroencephalography (EEG), but in some cases, the electrical hallmark of epilepsy might not be present interictally or if seizures are infrequent. The standard saying is “Treat the patient, not the EEG.” An EEG is a recording of the brain’s electrical activity and helps detect abnormal electrical activity, such as focal spikes or waves (consistent with focal epilepsy) or diffuse bilateral spikes and waves (consistent with generalized epilepsy).

Besides the main diagnostic tool of EEG, other tests such as a computed tomography scan (CT scan), magnetic resonance imaging (MRI), positron emission tomography (PET), single-photon emission computed tomography (SPECT), and genetic testing are necessary for a correct diagnosis as per the patient. Video EEG can help localize the ictal onset zone. In some cases, blood tests are also necessary, when the patients develop toxic and metabolic encephalopathies [13]. In high-volume epilepsy referral centers, an invasive evaluation or phase two is considered after formulating a reasonable hypothesis and using either subdural grids or stereoelectroencephalography (SEEG). Once an adequate number of seizures are obtained to identify the ictal onset zone, a surgical target can be identified for either surgical resection or laser ablation. If no favorable target is encountered, the person has multifocal epilepsy, or epilepsy originates in the eloquent areas, ablative surgery is not advised.

Treatment

The treatment objective is that patients do not develop seizures and have no side effects; however, this is not always possible. When patients are appropriately treated, they can live a normal life, although some patients are stigmatized and suffer discrimination [20].

When physicians understand epilepsy, epigenetic determinants, and pharmacogenomics, there is a high possibility of modifying or even thinking of curing epilepsy with pharmacological and non-pharmacological treatment strategies.

The main objective of antiepileptic drugs (AED) is to decrease the electrical activity of the brain by preventing neuronal depolarization. The main mechanisms include blocking sodium channels or calcium channels, enhancing potassium channel function, inhibiting excitation mediated by the neurotransmitter glutamate, or promoting inhibition mediated by GABA [3].

The ILAE has defined DRE as the failure of two adequate, well-tolerated drug trials and appropriately chosen and used AED as monotherapy or in combination to achieve sustained seizure freedom [21].

Nearly half of patients with newly diagnosed epilepsy achieve freedom from seizures with the first AED, 11% of patients with epilepsy become seizure-free after the second AED, and only 3% stop having seizures later on. The remaining 30-40% of epileptic patients are considered to have DRE [21,22].

Although is not fully understood, some risk factors favor the possibility of developing DRE, including age of onset, symptomatic epilepsy, abnormal EEG (with both slow wave and epileptiform discharges), status epilepticus, multiple seizure types, febrile seizure, abnormal neurologic image, intellectual disability, neurologic abnormality, status epilepticus, and psychiatric comorbidities [23,24].

When a patient has been diagnosed with DRE, an evaluation in a tertiary center is recommended to evaluate their candidacy for surgery. However, a significant proportion of patients cannot be treated with ablative surgery (resection or laser) due to comorbidities, the location of seizure focus in the eloquent cortex, the presence of multifocal epilepsy, or the inability to identify the ictal onset zone.

Neuromodulation

In patients for whom surgical resection or ablation is contraindicated, the electrical stimulation of the brain such as neuromodulation can be considered. Neuromodulation therapies are palliative non-pharmacologic alternatives where the nervous system’s activity in the body is modulated through non-invasive (e.g., transcranial magnetic stimulation or transcranial electric stimulation, such as transcranial alternating current stimulation) or invasive (e.g., vagal nerve stimulator and deep brain stimulation) methods [25,26].

Deep brain stimulation (DBS)

DBS is administered through a neurosurgical procedure to place an electrical medical device (neurostimulator) deep into the target site of the brain that functions like a “brain pacemaker.” This is done by delivering electric impulses via electrodes at specific deep nuclei to modulate the neural function and treat neurological (movement disorders and epilepsy) and psychiatric conditions [26].

Effective electrodes require several features such as biocompatibility, inertness, durability, stability over time, surgical feasibility, excellent conductivity, electrical properties, proper delivery of current, and spatial configuration. They are placed using stereotactic techniques with either head frame, frameless, or robotic techniques, and later attached to an implantable pulse generator (IPG) placed subfascially at the infraclavicular area. IPGs have two main functions: stimulation and, more recently, neural recording [25].

For controlling seizures, different regions of the brain have been investigated, including the cerebellum, centromedian thalamus, hippocampus, anterior nucleus of the thalamus, motor cortex, caudate, subthalamic nucleus, and other seizure foci [27].

DBS has revolutionized the treatment of neurological patients, and it has been used in other disorders such as depression, obsessive-compulsive disorder, and Alzheimer’s disease [28] with varying outcomes. Cluster headaches, addictions, anorexia, obesity, and Huntington’s disease have also been considered [29,30].

The success of this stimulatory device in Parkinson’s disease and essential tremor allowed the possibility of using it in patients with conditions such as refractory epilepsy [31].

The main purpose of DBS in patients with epilepsy is to modulate cortical excitability to reduce the frequency and severity of seizures.

Partial onset seizures are the most common seizure type in refractory epilepsy, and they frequently propagate along well-defined neural pathways. Networks such as the cortical-striatal-thalamic network and the limbic circuit of Papez provide nodes where electrical stimulation has the potential to regulate neural information, including abnormal signals that mediate seizure propagation. Thus, it is believed that these neural networks provide potential points for intervention and might interrupt the spread of seizure activity to the neocortex. However, such as in movement disorders, the exact mechanism of seizures control through DBS has not been fully elucidated [6,25,32].

Different targets of stimulation have been considered for the treatment with DBS, such as the anterior nucleus of the thalamus (ANT), centromedian nucleus, hippocampus, posterior hypothalamus, and cerebellum [6,27,33]. 

ANT has connections to the anterior cingulate cortex and orbitomedial prefrontal cortex and, hence, has a possible role in emotional and executive functions. Thus, sometimes, ANT DBS might exert psychiatrically adverse effects [34].

**Figure 1:**
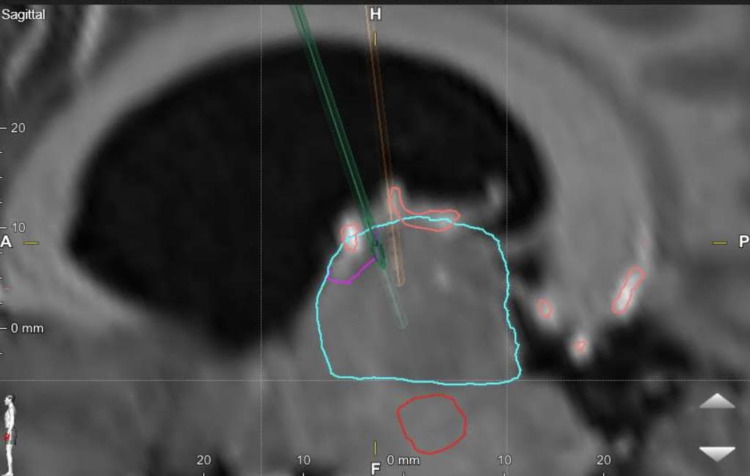
Sagittal view of anterior thalamic deep brain stimulation (DBS) The turquoise blue line represents the thalamus, while the purple line corresponds to ipsilateral anterior thalamic nucleus. No DBS contacts are within the ventricular system.

**Figure 2:**
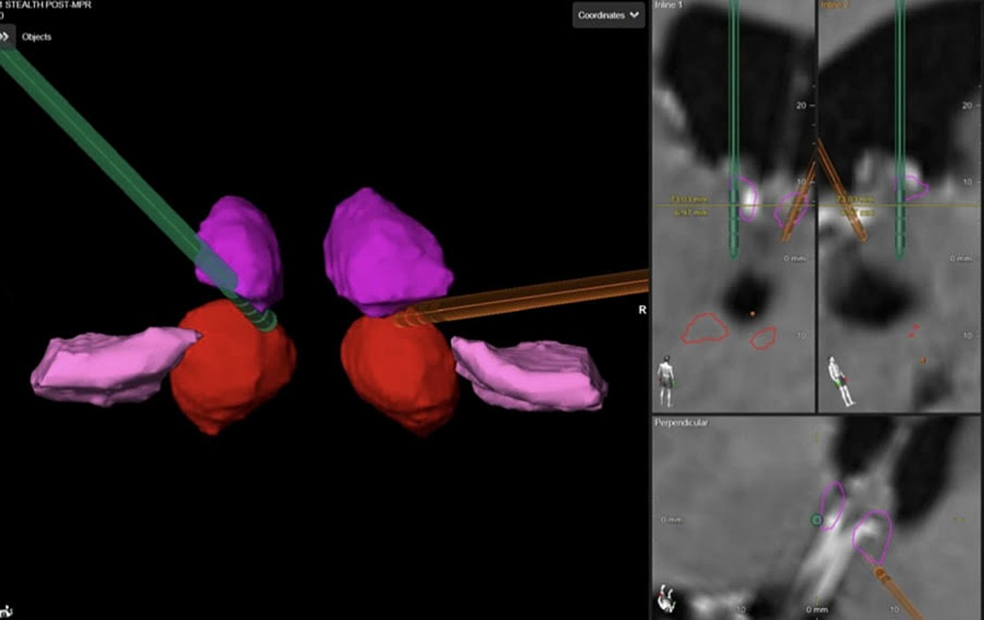
Tri-dimensional reconstruction of bilateral anterior thalamic deep brain stimulation (DBS) Ideally, the intraventricular DBS lead trajectory is preferred, although anatomical variations might require more lateral entry points (such as in this case with prominent thalamostriate venous angle, and superficially with prominent venous lacunae).

Applying DBS of the ATN has been recognized as an efficient treatment for DRE. However, targeting ATN is difficult, and up to 8% of lead misplacement has been reported [28]. There are targeting peculiarities to consider while performing ANT DBS. The nucleus is small, and MRI studies need to be relied on to visualize the target while considering thalamic anatomical variations, occasional unilateral atrophy, and mammillothalamic tract visualization and choose an adequate trajectory. This can be either transventricular trajectory, which is the ideal (Figure 1), versus lateral if the venous angle of the thalamostriate veins precludes an adequate angle of implantation. Overall, the significance of adequate planning is to keep adequate DBS lead contacts (at least two) inside the ANT (Figure 2).

To understand DBS on ANT, we consider it important to review the most important aspects of this nucleus.

ANT is a key component of the hippocampal system and part of the neural center of the thalamus, which has up to 60 nuclei. Each nucleus has a unique role and participates in the relay and integration of the different sensory signals, motor coordination, emotional activity, and various states of consciousness that transmit most information to the brain cortex. The nucleus also facilitates or inhibits projections to different lobes [2]. The anterior nuclear group occupies the superior region of the thalamus and is separated from the rest of the neural region by the internal medullary lamina in a Y-shaped lamina, which organizes all the nuclei in three subnuclei (anterior, medial, and lateral) with distinct connectivity with the subicular cortex, retrosplenial cortex, and mammillary bodies [34].

This nucleus is part of the limbic system and participates in the processing of emotions and recent memory. It receives afferent nerves from the hypothalamus through the mammillothalamic tract and projects its efferent nerves from the cingulate cortex [35].

The initial clinical series lesioning the thalamus for seizure control was done in 1967, supported by experimental involvement on seizure propagation by the Papez circuit [36]. In 1987, Cooper and Upton described bilateral ANT stimulation showing clinical control in a small case series [37]. Other subsequent small case series showed safety and efficacy of the ANT stimulation in drug-resistant epilepsy, describing minimal adverse events [38,39].

In this context, these results favored the design of the Stimulation of the Anterior Nucleus of the Thalamus for Epilepsy (SANTE) trial [40]. This study was a multicenter, randomized controlled, prospective, double-blind clinical trial of stimulation of the anterior nuclei of thalamus for epilepsy in 110 patients (18-65 years old). These subjects were diagnosed with partial and generalized seizures occurring at least six times per month but not more than 10 per day, as recorded in a three-month daily seizure diary. The baseline characteristics of the implanted participants who received electric stimuli were not different from those of the control group (age, years with epilepsy, seizures per month, prior surgical procedures for epilepsy, seizure types, location of seizure onset). Electrodes were implanted bilaterally in the ANT nucleus using a stereotactic technique.

One month after implantation, the participants were stimulated with 5 V (experimental group) or not stimulated (0 V, control group). After three months, patients of both the groups received stimulation from month four to month 13 in an unblinded phase. Limited stimulation parameter changes were allowed. At the end of month 13, participants were followed up in which AEDs and stimulation parameters could vary freely. Medications were kept constant during the three-month blinded phase and the nine-month unblinded phase. After stimulation, the mean seizure frequency stimulation was significantly less in the experimental group compared to the controls. This difference was greater over time. Complex partial seizures decreased more in the stimulated group, in comparison with the control group, during the blinded phase (36.3 vs. 12.1%; p = 0.041). The most severe seizure type improved 40% in the stimulated group but 20% in the control group (p = 0.047). Injuries caused by seizures were less in the stimulated group (7%) compared to the control group (26%; p = 0.01). For patients whose seizures originated in one or both temporal regions, the median seizure reduction in the stimulated group was greater in comparison with the control group (44.2% vs 21.8%, respectively). When the seizure origin was diffuse or multifocal, the reduction was 35% in the stimulated group versus 14.1% in the non-stimulated one. However, no difference in seizure reduction was observed when the origin was in the frontal, parietal, or occipital regions.

In a five-year unblinded follow-up study, only 75 patients continued with treatment, while 35 patients abandoned the treatment at some point of the study due to different reasons. Patients showed a 41% seizure reduction at the end of one year and 69% at the end of five years. Over the five years of follow-ups, 16% of patients had no seizures for at least six months [41].

According to the SANTE trial, the U.S. Food and Drug Administration approved DBS therapy for epilepsy in 2018, stating, “Bilateral stimulation of the anterior nucleus of the thalamus (ANT) for epilepsy is indicated as an adjunctive therapy for reducing the frequency of seizures in individuals 18 years of age or older diagnosed with epilepsy characterized by partial-onset seizures with or without secondary generalization that are refractory to three or more antiepileptic medications.”

After the publication of the SANTE trial, multiple additional open-label case series have been published [42,43]. Overall, these case reports support ANT DBS as a safe treatment option. In 2019, a prospective, randomized, double-blinded study in 18 patients with focal DRE reported that after comparing six months of stimulation with baseline, there was a 22% reduction in the frequency of seizures (p = 0.009), while four patients had ≥ 50% reduction in total seizure frequency and five patients ≥ 50% reduction in focal seizures after six months of stimulation, although no increased effect overtime was shown as in the SANTE trial [44].

Despite the interest and research evidence in ANT DBS for epilepsy, a clear understanding of the physiological mechanism, optimal programming, best targeting and lead placement techniques, ideal candidates and contraindications still is challenging [45,46].

## Conclusions

Currently, epilepsy can be treated with multiple options. Neuromodulation by deep brain stimulation is considered a safe and efficacious treatment for DRE when surgical resection or ablation is not considered adequate. The ANT is currently an FDA-approved target in the U.S.

There are high expectations on the results of DBS for epilepsy using ANT, although new long-term studies essential for evaluating clinical efficacy are lacking. There are too many variables that do not offer uniform results such as patient selection, targeting techniques that differ among institutions, use of microelectrode recording, intraoperative CT scan, intraoperative MRI, implantation by stereotactic frame, frameless, or robotic systems, stimulation parameters, directional leads, closed-loop systems, etc.

Overall, knowledge of real clinical applications, large-scale randomized trials, and long-term follow-up are needed to help further understand the mechanism of DBS and prove its efficacy, thereby increasing its implementation.

## References

[1] Kaculini CM, Tate-Looney AJ, Seifi A (2021). The history of epilepsy: from ancient mystery to modern misconception. Cureus.

[2] Wolf P (2014). History of epilepsy: nosological concepts and classification. Epileptic Disord.

[3] Stafstrom CE, Carmant L (2015). Seizures and epilepsy: an overview for neuroscientists. Cold Spring Harb Perspect Med.

[4] Fisher RS, van Emde Boas W, Blume W, Elger C, Genton P, Lee P, Engel J Jr (2005). Epileptic seizures and epilepsy: definitions proposed by the International League Against Epilepsy (ILAE) and the International Bureau for Epilepsy (IBE). Epilepsia.

[5] Fisher RS, Acevedo C, Arzimanoglou A (2014). ILAE official report: a practical clinical definition of epilepsy. Epilepsia.

[6] Rincon N, Barr D, Velez-Ruiz N (2021). Neuromodulation in drug resistant epilepsy. Aging Dis.

[7] Beghi E (2020). The epidemiology of epilepsy. Neuroepidemiology.

[8] Fiest KM, Sauro KM, Wiebe S (2017). Prevalence and incidence of epilepsy: A systematic review and meta-analysis of international studies. Neurology.

[9] Camfield P, Camfield C (2015). Incidence, prevalence and aetiology of seizures and epilepsy in children. Epileptic Disord.

[10] Harden C, Tomson T, Gloss D (2017). Practice guideline summary: sudden unexpected death in epilepsy incidence rates and risk factors: report of the Guideline Development, Dissemination, and Implementation Subcommittee of the American Academy of Neurology and the American Epilepsy Society. Epilepsy Curr.

[11] Thurman DJ, Begley CE, Carpio A (2018). The primary prevention of epilepsy: a report of the Prevention Task Force of the International League Against Epilepsy. Epilepsia.

[12] Sveinsson O, Andersson T, Carlsson S, Tomson T (2017). The incidence of SUDEP: a nationwide population-based cohort study. Neurology.

[13] Anwar H, Khan QU, Nadeem N, Pervaiz I, Ali M, Cheema FF (2020). Epileptic seizures. Discoveries (Craiova).

[14] Hamani C, Hodaie M, Lozano AM (2005). Present and future of deep brain stimulation for refractory epilepsy. Acta Neurochir (Wien).

[15] Wu C, Sharan AD (2013). Neurostimulation for the treatment of epilepsy: a review of current surgical interventions. Neuromodulation.

[16] Zangiabadi N, Ladino LD, Sina F, Orozco-Hernández JP, Carter A, Téllez-Zenteno JF (2019). Deep brain stimulation and drug-resistant epilepsy: a review of the literature. Front Neurol.

[17] Lega BC, Halpern CH, Jaggi JL, Baltuch GH (2010). Deep brain stimulation in the treatment of refractory epilepsy: update on current data and future directions. Neurobiol Dis.

[18] Kariuki SM, Ngugi AK, Kombe MZ (2021). Prevalence and mortality of epilepsies with convulsive and non-convulsive seizures in Kilifi, Kenya. Seizure.

[19] Subota A, Khan S, Josephson CB (2019). Signs and symptoms of the postictal period in epilepsy: a systematic review and meta-analysis. Epilepsy Behav.

[20] Fernandes PT, Snape DA, Beran RG, Jacoby A (2011). Epilepsy stigma: what do we know and where next?. Epilepsy Behav.

[21] Kwan P, Arzimanoglou A, Berg AT (2010). Definition of drug resistant epilepsy: consensus proposal by the ad hoc Task Force of the ILAE Commission on Therapeutic Strategies. Epilepsia.

[22] Chen Z, Brodie MJ, Liew D, Kwan P (2018). Treatment outcomes in patients with newly diagnosed epilepsy treated with established and new antiepileptic drugs: a 30-year longitudinal cohort study. JAMA Neurol.

[23] Guery D, Rheims S (2021). Clinical management of drug resistant epilepsy: a review on current strategies. Neuropsychiatr Dis Treat.

[24] Xue-Ping W, Hai-Jiao W, Li-Na Z, Xu D, Ling L (2019). Risk factors for drug-resistant epilepsy: a systematic review and meta-analysis. Medicine (Baltimore).

[25] Wu YC, Liao YS, Yeh WH, Liang SF, Shaw FZ (2021). Directions of deep brain stimulation for epilepsy and Parkinson's disease. Front Neurosci.

[26] Eastin TM, Lopez-Gonzalez MA (2017). Stimulation and neuromodulation in the treatment of epilepsy. Brain Sci.

[27] Fisher RS, Velasco AL (2014). Electrical brain stimulation for epilepsy. Nat Rev Neurol.

[28] Lozano AM, Fosdick L, Chakravarty MM (2016). A Phase II Study of Fornix Deep Brain Stimulation in Mild Alzheimer's Disease. J Alzheimers Dis.

[29] Pycroft L, Stein J, Aziz T (2018). Deep brain stimulation: an overview of history, methods, and future developments. Brain Neurosci Adv.

[30] Dandekar MP, Fenoy AJ, Carvalho AF, Soares JC, Quevedo J (2018). Deep brain stimulation for treatment-resistant depression: an integrative review of preclinical and clinical findings and translational implications. Mol Psychiatry.

[31] Halpern C, Hurtig H, Jaggi J, Grossman M, Won M, Baltuch G (2007). Deep brain stimulation in neurologic disorders. Parkinsonism Relat Disord.

[32] Laxpati NG, Kasoff WS, Gross RE (2014). Deep brain stimulation for the treatment of epilepsy: circuits, targets, and trials. Neurotherapeutics.

[33] Velasco AL, Velasco F, Jiménez F (2006). Neuromodulation of the centromedian thalamic nuclei in the treatment of generalized seizures and the improvement of the quality of life in patients with Lennox-Gastaut syndrome. Epilepsia.

[34] Järvenpää S, Lehtimäki K, Rainesalo S, Möttönen T, Peltola J (2020). Improving the effectiveness of ANT DBS therapy for epilepsy with optimal current targeting. Epilepsia Open.

[35] Tassigny D, Soler-Rico M, Delavallée M, Santos SF, El Tahry R, Raftopoulos C (2020). Anterior thalamic nucleus deep brain stimulation for refractory epilepsy: preliminary results in our first 5 patients. Neurochirurgie.

[36] Mullan S, Vailati G, Karasick J, Mailis M (1967). Thalamic lesions for the control of epilepsy. A study of nine cases. Arch Neurol.

[37] Upton AR, Amin I, Garnett S, Springman M, Nahmias C, Cooper IS (1987). Evoked metabolic responses in the limbic-striate system produced by stimulation of anterior thalamic nucleus in man. Pacing Clin Electrophysiol.

[38] Hodaie M, Wennberg RA, Dostrovsky JO, Lozano AM (2002). Chronic anterior thalamus stimulation for intractable epilepsy. Epilepsia.

[39] Kerrigan JF, Litt B, Fisher RS (2004). Electrical stimulation of the anterior nucleus of the thalamus for the treatment of intractable epilepsy. Epilepsia.

[40] Fisher R, Salanova V, Witt T (2010). Electrical stimulation of the anterior nucleus of thalamus for treatment of refractory epilepsy. Epilepsia.

[41] Salanova V, Witt T, Worth R (2015). Long-term efficacy and safety of thalamic stimulation for drug-resistant partial epilepsy. Neurology.

[42] Lee KJ, Shon YM, Cho CB (2012). Long-term outcome of anterior thalamic nucleus stimulation for intractable epilepsy. Stereotact Funct Neurosurg.

[43] Oh YS, Kim HJ, Lee KJ, Kim YI, Lim SC, Shon YM (2012). Cognitive improvement after long-term electrical stimulation of bilateral anterior thalamic nucleus in refractory epilepsy patients. Seizure.

[44] Herrman H, Egge A, Konglund AE, Ramm-Pettersen J, Dietrichs E, Taubøll E (2019). Anterior thalamic deep brain stimulation in refractory epilepsy: a randomized, double-blinded study. Acta Neurol Scand.

[45] Montgomery EB Jr (2012). The epistemology of deep brain stimulation and neuronal pathophysiology. Front Integr Neurosci.

[46] Kaufmann E, Bartolomei F, Boon P (2020). European expert opinion on ANT-DBS therapy for patients with drug-resistant epilepsy (a Delphi consensus). Seizure.

